# Supplementary material for: Associations of Fecal Microbiota with Ectopic Fat in African Caribbean Men
Source: Microorganisms. 2024 Apr 17;12(4):812. doi: 10.3390/microorganisms12040812 (PMC11052294; doi:10.3390/microorganisms12040812)
Supplement: Supplementary file 1 [file microorganisms-12-00812-s001.zip › microorganisms-2957544-supplementary.pdf]

SUPPLEMENTARY MATERIALS

MANUSCRIPT TITLE: Associations of Fecal Microbiota with Ectopic Fat in African-Caribbean Men

AUTHORS: Curtis Tilves, Noel T. Mueller, Joseph M. Zmuda, Allison L Kuipers, Barbara Methe, Kelvin Li, John Jeffrey Carr, James G Terry, Victor Wheeler, Sangeeta Nair, Iva Miljkovic

CORRESPONDING AUTHOR CONTACT INFO:

Name: Curtis Tilves

Postal Address: 1890 N Revere Ct, Suite 1002, 1st Floor, Mail Stop F426, Aurora, CO 80045

E-mail Address: curtis.tilves@cuanschutz.edu

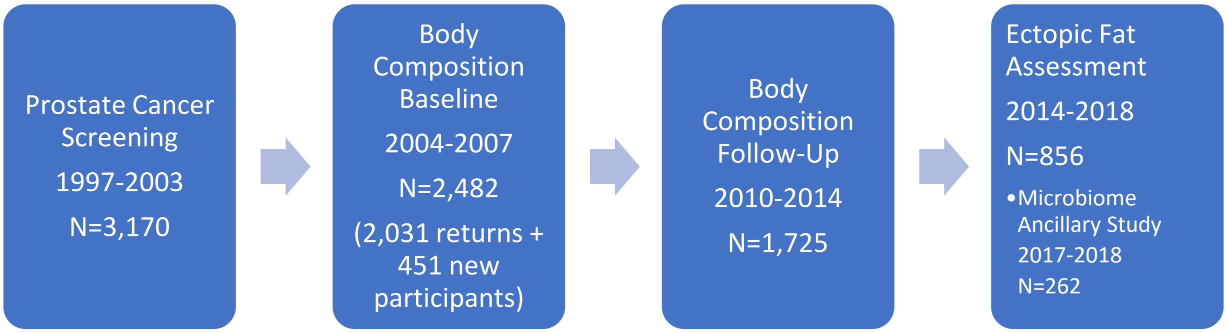

Supplementary Figure S1 [title]: *Study Flow Chart for the Tobago Health Study.*
Supplementary Figure S1 [legend]: *The first visit of the Tobago Health Study occurred between 1997-2003, which recruited men 40+ years who were ambulatory and not terminally ill. Between 2004-2007, men were invited to return for a (non-CT) body composition assessment visit; at this time, new participant recruitment also occurred using the same inclusion criteria as the first visit. Between 2010-2014, men were again invited to return for additional (non-CT) body composition measurements at a third study visit. A fourth study visit occurred between 2014-2018, in which a convenience sample of those attending the third study visit were invited for CT body composition measurements. Within this fourth study visit (between 2017-2018), we additionally implemented a fecal sample collection for microbiome assessment among a convenience sample of N=262 men who had already underwent CT body composition measurements.*

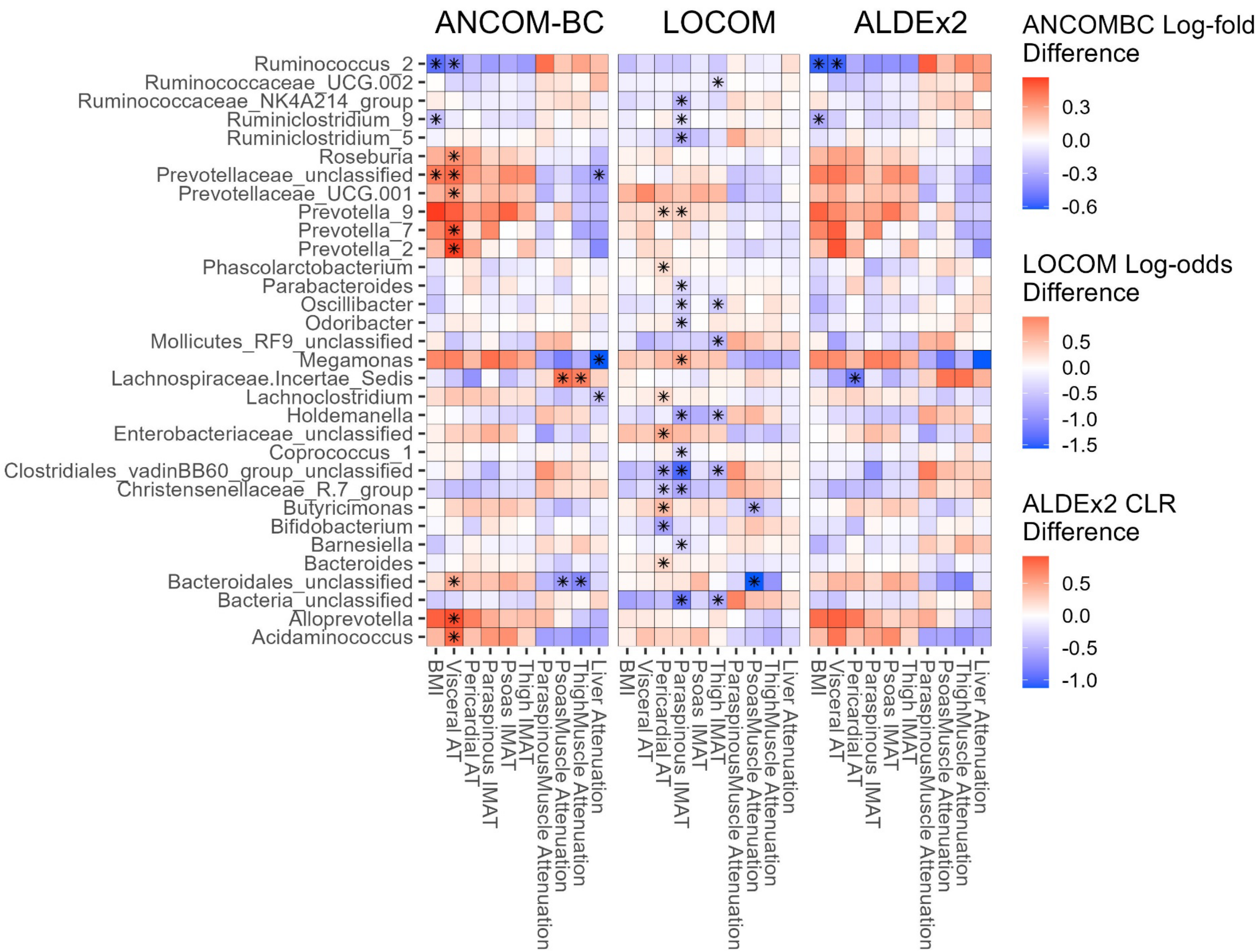
ANCOM-BC
LOCOM
ALDEx2
ANCOMBC Log-fold Difference
0.3
0.0
-0.3
-0.6
LOCOM Log-odds Difference
0.5
0.0
-0.5
-1.0
-1.5
ALDEx2 CLR Difference
0.5
0.0
-0.5
-1.0
Ruminococcus_2
Ruminococcaceae_UCG.002
Ruminococcaceae_NK4A214_group
Ruminiclostridium_9
Ruminiclostridium_5
Roseburia
Prevotellaceae_unclassified
Prevotellaceae_UCG.001
Prevotella_9
Prevotella_7
Prevotella_2
Phascolarctobacterium
Parabacteroides
Oscillibacter
Odoribacter
Mollicutes_RF9_unclassified
Megamonas
Lachnospiraceae.Incertae_Sedis
Lachnoclostridium
Holdemanella
Enterobacteriaceae_unclassified
Coprococcus_1
Clostridiales_vadinBB60_group_unclassified
Christensenellaceae_R.7_group
Butyricimonas
Bifidobacterium
Barnesiella
Bacteroides
Bacteroidales_unclassified
Bacteria_unclassified
Alloprevotella
Acidaminococcus
BMI
Visceral AT
Pericardial AT
Paraspinous IMAT
Psoas IMAT
Thigh IMAT
ParaspinousMuscle Attenuation
PsoasMuscle Attenuation
ThighMuscle Attenuation
Liver Attenuation

Supplementary Figure S2 [title]: *Estimated changes in OTU abundance per standard deviation increment in BMI or fat, according to three differential abundance statistical approaches, among men from the Tobago Health Study.*

Supplementary Figure S2 [legend]: *A total of 193 men were included in the analyses. Rare (prevalence of <10%) and low-abundant (mean relative abundance <0.1%) microbes were pre-filtered from samples. Statistical models were analyzed separately for each BMI or fat measure; models adjusted for age (years), educational attainment (primary, secondary, or post-secondary), hours walked per week for exercise (hours), current smoking status (yes vs. no), drinking 4 or more alcoholic drinks per week (yes vs. no), and time difference between CT scans and fecal sample collection (years). Asterisks (*) denote statistically significantly associated OTUs, with different FDR-corrected q-value thresholds according to the model used (ANCOM-BC: q < 0.05; LOCOM and ALDEx2: q < 0.20). OTUs associated with at least one BMI or fat measure in at least one differential abundance testing model are included.*

Note: differential abundance models use different approaches (ex: linear regression, logistic regression) and different scaling of outcomes; thus, comparisons by strength of association across approaches is not appropriate. However, comparisons of directions of association, i.e., the heatmap colors, can be made.

(Left): Heatmap of ANCOM-BC results, showing the log-fold difference in microbial abundance per standard deviation increment in BMI or fat measure. (Middle): Heatmap of LOCOM results, showing the log-odds difference in microbial abundance per standard deviation increment in BMI or fat measure. (Right): Heatmap of ALDEx2 results, showing the CLR difference in microbial abundance per standard deviation increment in BMI or fat measure.

Abbreviations: OTU = operational taxonomic unit, ANCOM-BC = Analysis of Compositions of Microbiomes with Bias Correction, FDR = false discovery rate, IMAT = intermuscular fat, SD = standard deviation, LOCOM = logistic compositional analysis, CLR = center logratio

Supplementary Table S1: Sample characteristics comparing the N=262 men who returned for the Microbiome Visit compared to the remaining N=549 who did not, from the Tobago Cohort Study.

Characteristic	Didn't Return for Microbiome Visit (N=594)	Returned for Microbiome Visit (N=262)	p-value
**Age (years)**	63.0 [57.0, 71.0]	60.5 [56.3, 68.0]	0.003
**African Caribbean Ethnicity**	545 (91.8%)	236 (90.1%)	0.999
Missing	6 (1.0%)	8 (3.1%)	
**Educational Attainment**			
Primary	423 (71.2%)	179 (68.3%)	0.337
Post-Secondary	80 (13.5%)	29 (11.1%)	
Secondary	81 (13.6%)	44 (16.8%)	
Missing	10 (1.7%)	10 (3.8%)	
**Hours Walked per Week**	1.5 [0.0, 4.5]	2.0 [0.0, 5.6]	0.546
Missing	7 (1.2%)	3 (1.1%)	
**Drinks 4+ alcohol/week (yes)**	81 (13.6%)	31 (11.8%)	0.511
**Current Smoking Status (yes)**	42 (7.1%)	26 (9.9%)	0.171
Missing	1 (0.2%)	0 (0%)	
**BMI (kg/m^2)**	27.0 [24.3, 30.3]	27.7 [25.0, 30.4]	0.074
Missing	2 (0.3%)	1 (0.4%)	
**Alternative Mediterranean Diet Score (range 1-9)**	4.0 [3.0, 5.0]	4.0 [3.0, 5.0]	0.209
Missing	215 (36.2%)	49 (18.7%)	
**Energy intake (kcal/day)**	2497.1 [1839.6, 3195.3]	2338.4 [1755.4, 3016.0]	0.141
Missing	215 (36.2%)	49 (18.7%)	

Supplementary Table S5 [Footnote]: *Sample descriptive statistics are provided for N=856 men from the Tobago Cohort Study who did (N=262) or did not (N=594) attend the ancillary microbiome study visit. Characteristics are presented as median (interquartile range) or N (%). P-values were calculated based on a Kruskal-Wallis or Fisher's exact test.*

Supplementary Table S2: Age-adjusted partial Pearson correlations of BMI and fat measures, among men from the Tobago Health Study

	VAT	Pericardial fat	Paraspinous IMAT	Psoas IMAT	Thigh IMAT	Paraspinous muscle attenuation	Psoas muscle attenuation	Thigh muscle attenuation	Liver Attenuation
BMI	0.67 †	0.59 †	0.49 †	0.44 †	0.75 †	-0.26 †	-0.21 †	-0.31 †	-0.51 †
VAT		0.69 †	0.37 †	0.49 †	0.61 †	-0.19 †	-0.22 †	-0.31 †	-0.45 †
Pericardial fat			0.39 †	0.39 †	0.57 †	-0.20 †	-0.32 †	-0.41 †	-0.32 †
Paraspinous IMAT				0.51 †	0.57 †	-0.69 †	-0.46 †	-0.50 †	-0.19 †
Psoas IMAT					0.45 †	-0.39 †	-0.50 †	-0.39 †	-0.23 †
Thigh IMAT						-0.37 †	-0.35 †	-0.54 †	-0.33 †
Paraspinous muscle attenuation							0.58 †	0.65 †	0.12
Psoas muscle attenuation								0.72 †	0.15 †
Thigh muscle attenuation									0.14 †

†: significant at p<0.05

Supplementary Table S2 [Footnote]: *A total of N=193 men were included in the analyses. Partial Pearson correlations with age adjustment were calculated. Statistically significant correlations at p<0.05 is indicated with (†). Note: lower muscle or liver attenuation reflects greater lipid accumulation.*
Abbreviations: VAT = visceral fat, IMAT = intermuscular fat

Supplementary Table S3: Age-by-BMI or age-by-fat interaction term p-values in alpha diversity models, among men from the Tobago Health Study

BMI or Fat Measure	Observed OTUs	Pielou's Evenness	Shannon Diversity Index
BMI	0.2759	0.7461	0.9264
VAT	0.0953	0.7697	0.2981
Pericardial fat	0.0844	0.1242	0.8736
Paraspinous IMAT	0.7303	0.5460	0.5146
Psoas IMAT	**0.0287**	0.9605	0.3816
Thigh IMAT	0.1230	0.9219	0.5107
Paraspinous muscle attenuation	0.8362	0.2403	0.6981
Psoas muscle attenuation	0.5668	**0.0492**	0.4218
Thigh muscle attenuation	**0.0295**	0.1434	0.8573
Liver Attenuation	0.7953	0.4329	0.3042

Supplementary Table S3 [Footnote]: *A total of N=193 men were included in the analyses. P-values for interaction terms from models are presented. Statistically significant interactions at p<0.05 are indicated with bold text. Multivariable models included age (years), educational attainment (primary, secondary, or post-secondary), hours walked per week for exercise (hours), current smoking status (yes vs. no), drinking 4 or more alcoholic drinks per week (yes vs. no), time difference between CT scans and fecal sample collection (years), unrarefied sequencing depth (in observed OTU models), and an interaction between age and alpha diversity metric.*
Abbreviations: VAT = visceral fat, IMAT = intermuscular fat, OTU = operational taxonomic unit

Supplementary Table S4: Absolute difference in regression coefficients for fat measure predictors after adjustment for muscle volumes, among men from the Tobago Health Study

BMI or Fat Measures	Observed OTUs	Pielou's Evenness	Shannon Diversity Index
Pericardial fat	0.01	0.00	0.00
Paraspinous IMAT	0.00	0.00	-0.01
Psoas IMAT	0.00	-0.03	-0.02
Thigh IMAT	0.01	-0.03	-0.02
Paraspinous muscle attenuation	0.01	0.00	-0.01
Psoas muscle attenuation	0.01	-0.02	-0.01
Thigh muscle attenuation	0.00	-0.01	-0.01

Supplementary Table S4 [Footnote]: *A total of N=193 men were included in the analyses. Fecal microbiota samples were first rarefied to a sequencing depth of 1,500 reads. Differences in regression coefficients (modeled as per SD increase in alpha diversity metric) were calculated as* $\beta_{\text{not muscle volume adjusted}} - \beta_{\text{muscle volume adjusted}}$*. Multivariable models included age (years), educational attainment (primary, secondary, or post-secondary), hours walked per week for exercise (hours), current smoking status (yes vs. no), drinking 4 or more alcoholic drinks per week (yes vs. no), time difference between CT scans and fecal sample collection (years), and unrarefied sequencing depth (in observed OTU models); in muscle volume adjusted models, the respective muscle volume (cardiac, paraspinous, psoas, thigh) was additionally adjusted.*

Abbreviations: VAT = visceral fat, IMAT = intermuscular fat, OTU = operational taxonomic unit

Supplementary Table S5: Difference in adjusted R^2 from PERMANOVA models for fat measure predictors after adjustment for muscle volumes, among men from the Tobago Health Study

Fat Measure	ΔR^2
Pericardial fat	0.01%
Paraspinous IMAT	-0.01%
Psoas IMAT	0.27%
Thigh IMAT	0.30%
Paraspinous muscle attenuation	-0.03%
Psoas muscle attenuation	0.13%
Thigh muscle attenuation	-0.02%

Supplementary Table S5 [Footnote]: *A total of N=193 men were included in the analyses. Fecal microbiota samples were first rarefied to a sequencing depth of 1,500 reads. Differences in adjusted R^2 were calculated as $R^2_{\text{not muscle volume adjusted}} - R^2_{\text{muscle volume adjusted}}$. Multivariable models included age (years), educational attainment (primary, secondary, or post-secondary), hours walked per week for exercise (hours), current smoking status (yes vs. no), drinking 4 or more alcoholic drinks per week (yes vs. no), and time difference between CT scans and fecal sample collection (years); in muscle volume adjusted models, the respective muscle volume (cardiac, paraspinous, psoas, thigh) was additionally adjusted. Abbreviations: PERMANOVA = permutational analysis of variance, IMAT = intermuscular fat, CT = computed tomography*

Supplementary Table S6: Absolute difference in regression coefficients for BMI or fat measure predictors after adjustment for diet (N=164), among men from the Tobago Health Study

	Not Fat and Diet Co-Adjusted			Fat and Diet Co-Adjusted			$\beta_{not\ diet\text{-}adjusted} - \beta_{diet\text{-}adjusted}$		
BMI or Fat Measures	Observed OTUs	Pielou's Evenness	Shannon Diversity Index	Observed OTUs	Pielou's Evenness	Shannon Diversity Index	Observed OTUs	Pielou's Evenness	Shannon Diversity Index
BMI	-0.02	-0.15	-0.13	-0.02	-0.15	-0.13	-0.01	0.00	0.00
VAT	-0.13	-0.20	-0.19	-0.13	-0.21	-0.20	0.00	0.01	0.01
Pericardial fat	-0.04	-0.13	-0.12	-0.05	-0.14	-0.13	0.01	0.01	0.01
Paraspinous IMAT	-0.02	-0.08	-0.07	-0.03	-0.09	-0.08	0.00	0.01	0.01
Psoas IMAT	-0.01	-0.05	-0.05	-0.01	-0.06	-0.05	0.00	0.00	0.00
Thigh IMAT	-0.06	-0.11	-0.10	-0.05	-0.11	-0.10	-0.01	0.00	0.00
Paraspinous muscle attenuation	0.01	0.01	0.01	0.01	0.02	0.02	0.00	-0.01	-0.01
Psoas muscle attenuation	0.03	0.01	0.01	0.04	0.01	0.02	0.00	-0.01	-0.01
Thigh muscle attenuation	0.03	0.04	0.04	0.04	0.06	0.05	0.00	-0.02	-0.01
Liver Attenuation	0.10	0.15	0.14	0.07	0.13	0.13	0.03	0.01	0.02

Supplementary Table S6 [Footnote]: *A total of N=164 men with dietary intake data were included in the analyses. Differences in regression coefficients (modeled as per SD increase in alpha diversity metric) were calculated as* $\beta_{not\ diet\text{-}adjusted} - \beta_{diet\text{-}adjusted}$*. Multivariable models included age (years), educational attainment (primary, secondary, or post-secondary), hours walked per week for exercise (hours), current smoking status (yes vs. no), drinking 4 or more alcoholic drinks per week (yes vs. no), time difference between CT scans and fecal sample collection (years), and unrarefied sequencing depth (in observed OTU models); models with diet additionally adjusted for an alternative Mediterranean diet score and total energy intake (kcal/day).*

†: alternative Mediterranean diet score and energy intake were both included in any diet-adjusted models. Change in β for diet measures is reported for BMI and VAT models, but was similar across all models.

Abbreviations: VAT = visceral fat, IMAT = intermuscular fat, OTU = operational taxonomic unit

Supplementary Table S7: Difference in % variance (adjusted R^2) explained by fat or diet predictors from PERMANOVA models, before and after co-adjustment of diet and fat measures (N=164), among men from the Tobago Health Study

BMI, Fat, or Diet Measures	% Variance Explained, No Diet and Fat Co-Adjustment	% Variance Explained, With Fat and Diet Co-adjustment	Δ R^2
BMI	2.266%	2.301%	-0.04%
VAT	2.439%	2.568%	-0.13%
Pericardial fat	1.560%	1.686%	-0.13%
Paraspinous IMAT	0.900%	1.016%	-0.12%
Psoas IMAT	0.707%	0.728%	-0.02%
Thigh IMAT	0.832%	0.844%	-0.01%
Paraspinous muscle attenuation	0.358%	0.380%	-0.02%
Psoas muscle attenuation	0.247%	0.233%	0.01%
Thigh muscle attenuation	0.568%	0.707%	-0.14%
Liver Attenuation	1.032%	0.961%	0.07%
Alternative Mediterranean Diet Score †	0.543%	BMI Model: 0.522%<br>VAT Model: 0.597%	BMI Model: -0.02%<br>VAT Model: -0.05%
Energy Intake †	0.615%	BMI Model: 0.649%<br>VAT Model: 0.637%	BMI Model: -0.03%<br>VAT Model: -0.02%

Supplementary Table S7 [Footnote]: *A total of N=164 men with dietary intake data were included in the analyses. Differences in adjusted* R^2 *were calculated as* $R^2_{not\ diet\text{-}adjusted} - R^2_{diet\text{-}adjusted}$. *Multivariable models included age (years), educational attainment (primary, secondary, or post-secondary), hours walked per week for exercise (hours), current smoking status (yes vs. no), drinking 4 or more alcoholic drinks per week (yes vs. no), and time difference between CT scans and fecal sample collection (years); models with diet additionally adjusted for an alternative Mediterranean diet score and total energy intake (kcal/day).*
†: alternative Mediterranean diet score and energy intake were both included in any diet-adjusted models. Change in % variance explained by diet is reported for BMI and VAT models, but was similar across all models.
Abbreviations: VAT = visceral fat, IMAT = intermuscular fat, OTU = operational taxonomic unit

Supplementary Table S8: β (95% CI) for the association of diet measures with a 1 standard deviation larger BMI, fat measures, and microbiome alpha diversity measures (N=164), among men from the Tobago Health Study

Outcomes: BMI, Fat, or Alpha Diversity Measures	Predictor: Alternative Mediterranean Diet Score (per 1-unit increase) †	Predictor: Energy Intake (per 500 kcal) †
BMI	0.01 (-0.08, 0.10)	-0.03 (-0.13, 0.07)
VAT	-0.02 (-0.12, 0.07)	-0.04 (-0.14, 0.06)
Pericardial fat	-0.05 (-0.14, 0.05)	-0.02 (-0.12, 0.09)
Paraspinous IMAT	-0.04 (-0.13, 0.05)	-0.04 (-0.14, 0.05)
Psoas IMAT	-0.01 (-0.10, 0.09)	-0.01 (-0.11, 0.09)
Thigh IMAT	0.02 (-0.08, 0.12)	-0.05 (-0.15, 0.06)
Paraspinous muscle attenuation	0.04 (-0.04, 0.12)	0.07 (-0.02, 0.15)
Psoas muscle attenuation	0.02 (-0.07, 0.11)	0.04 (-0.06, 0.14)
Thigh muscle attenuation	0.05 (-0.04, 0.14)	0.06 (-0.03, 0.16)
Liver Attenuation	-0.10 (-0.19, -0.00)	0.08 (-0.03, 0.18)
Observed OTUs	-0.09 (-0.19, 0.00)	0.08 (-0.03, 0.18)
Pielou's Evenness	-0.06 (-0.16, 0.04)	-0.01 (-0.11, 0.10)
Shannon Diversity Index	-0.07 (-0.17, 0.02)	0.01 (-0.09, 0.12)

Supplementary Table S8 [Footnote]: *A total of N=164 men with dietary intake data were included in the analyses. Multivariable linear regression models included adjustment for Alternative Mediterranean Diet score, energy intake (per 500 kcal/day), age (years), educational attainment (primary, secondary, or post-secondary), hours walked per week for exercise (hours), current smoking status (yes vs. no), drinking 4 or more alcoholic drinks per week (yes vs. no), time difference between CT scans and fecal sample collection (years), and unrarefied sequencing depth (in observed OTU model). Outcomes were standardized measures of BMI, fat, or alpha diversity.* ***Bold text*** *indicates a statistically significant association at p<0.05*
